# Identification of key structural elements for neuronal calcium sensor-1 function in the regulation of the temperature-dependency of locomotion in C. elegans

**DOI:** 10.1186/1756-6606-6-39

**Published:** 2013-08-27

**Authors:** Victoria M Martin, James R Johnson, Lee P Haynes, Jeff W Barclay, Robert D Burgoyne

**Affiliations:** 1 Department of Cellular and Molecular Physiology, The Physiological Laboratory, Institute of Translational Medicine, University of Liverpool, Liverpool, L69 3BX, UK

**Keywords:** Calcium signalling, Calcium binding proteins, C. elegans, NCS-1

## Abstract

**Background:**

Intracellular Ca^2+^ regulates many aspects of neuronal function through Ca^2+^ binding to EF hand-containing Ca^2+^ sensors that in turn bind target proteins to regulate their function. Amongst the sensors are the neuronal calcium sensor (NCS) family of proteins that are involved in multiple neuronal signalling pathways. Each NCS protein has specific and overlapping targets and physiological functions and specificity is likely to be determined by structural features within the proteins. Common to the NCS proteins is the exposure of a hydrophobic groove, allowing target binding in the Ca^2+^-loaded form. Structural analysis of NCS protein complexes with target peptides has indicated common and distinct aspects of target protein interaction. Two key differences between NCS proteins are the size of the hydrophobic groove that is exposed for interaction and the role of their non-conserved C-terminal tails.

**Results:**

We characterised the role of NCS-1 in a temperature-dependent locomotion assay in *C. elegans* and identified a distinct phenotype in the *ncs-1* null in which the worms do not show reduced locomotion at actually elevated temperature. Using rescue of this phenotype we showed that NCS-1 functions in AIY neurons. Structure/function analysis introducing single or double mutations within the hydrophobic groove based on information from characterised target complexes established that both N- and C-terminal pockets of the groove are functionally important and that deletion of the C-terminal tail of NCS-1 did not impair its ability to rescue.

**Conclusions:**

The current work has allowed physiological assessment of suggestions from structural studies on the key structural features that underlie the interaction of NCS-1 with its target proteins. The results are consistent with the notion that full length of the hydrophobic groove is required for the regulatory interactions underlying NCS-1 function whereas the C-terminal tail of NCS-1 is not essential. This has allowed discrimination between two potential modes of interaction of NCS-1 with its targets.

## Background

The neuronal calcium sensor (NCS) proteins form a family of Ca^2+^-binding proteins which have been implicated in the control of multiple aspects of neuronal function [1-3]. In mammals the family is encoded by 14 genes with multiple splice variants increasing the overall complexity of the family [3]. Amongst the family members NCS-1, the neurocalcins/VILIPs (together the visinin-like proteins), and the KChIPs (K^+^ channel interacting proteins) are expressed in various neurons of the nervous system whereas recoverin, and the GCAPs (guanylate cyclase activating proteins) are predominantly or solely expressed in the retina [4,5]. Whilst being similar to calmodulin they are distinguished by usually having a higher affinity for Ca^2+^ binding and certain family members are membrane targeted through N-terminal myristoylation (NCS-1, VILIPs, recoverin, and KChIP1) or palmitoylation (certain KChIPs). Some NCS proteins display reversible membrane association through a unique Ca^2+^ /myristoyl switch mechanism [6-9]. Despite having high levels of sequence and structural similarity the NCS proteins have a number of known non-redundant functions [3,10].

NCS-1, the primordial member of the NCS family, has orthologues from *Saccharomyces cerevisiae* (Frq1) [11] to man and has been implicated in several neuronal functions including regulation of neurotransmitter release [12,13], membrane traffic [14], voltage gated Ca^2+^ channels [15-17], neuronal development [18,19], synaptic plasticity [20,21] and learning [22,23]. NCS-1 is N-terminally myristoylated which allows its association with the plasma membrane and the trans-Golgi network [7] and it cycles between membrane and cytosolic pools [24]. NCS-1 is known to interact with a wide range of potential target proteins [25,26] including phosphatidylinositol-4-kinase (PI4K) IIIβ [14,27] and its orthologue Pik1 in yeast [11], ARF1 [14,28], interleukin receptor accessory protein like-1 (IL1RAPL1) [29], TRPC5 channels [18], InsP(3) receptors [30] and dopamine D2 and D3 receptors [31]. Studies at an organism level have identified key NCS-1 functions [3,10]. In the mouse, for example, NCS-1 has been implicated in exploratory behaviour and in the acquisition of spatial memory by regulating the surface expression of dopamine D2 receptors in the hippocampal dentate gyrus [23]. In C. elegans NCS-1 is expressed in sensory neurons and is involved in neuronal pathways that control long term memory for thermosensation [22] and has also been implicated in chemotaxis [32].

The specific functions of the NCS proteins are likely to be determined predominantly by interactions with specific target proteins [3,10]. Structural studies have characterised several of the NCS proteins revealing that that have very similar main chain topologies [33] and they have in common the exposure of a hydrophobic groove in the Ca^2+^-loaded form [6,34-40]. Structural data is available for complexes of recoverin with an N-terminal fragment of rhodopsin kinase [41], KChIP1 with an N-terminal region of the Kv4.3 potassium channel [38,39], orthologues of NCS-1 (Frq1) in *Saccharomyces cerevisiae* and Schizo*saccharomyces pombe* with fragments of Pik1 the orthologue of phosphatidylinositol-4-kinase (PI4K)IIIβ [42,43] and a peptide from the C-terminus of the dopamine D2 receptor with human NCS-1 [44]. Specificity of target interaction has been suggested to be due to the varying size and shape of the hydrophobic groove, differences in distribution in surrounding charged residues and interactions of the extreme C-terminus of the proteins [10,33]. Within the complexes there are two main modes of interaction with the hydrophobic groove that have been observed. In the first mode (recoverin and KChIP1) a helix from the target is bound to the N-terminal pocket of the hydrophobic groove and the C-terminus of the NCS protein occludes the C-terminal pocket of the groove [41], [38,39], where it can make direct contact with the target [45]. In the second mode (Frq1) two helices from the target interact across the entire exposed hydrophobic groove. A similar mode of interaction has been suggested for mammalian NCS-1 [40,44] based on the apparent exposure of the whole of the hydrophobic groove in the crystal structure of NCS-1. In contrast, however, a recent study that solved the NMR solution structure of human NCS-1 showed that the C-terminal tail of NCS-1 occludes the C-terminal part of the groove [46].

The molecular assessment of NCS proteins interactions has been based on structural studies that have used only short fragments of target proteins. The physiological significance for NCS-1 of each of the modes of interaction *in vivo* has been unclear. We have now established a key role for NCS-1 in regulation of temperature-dependent locomotion in *Caenorhabditis elegans* and used this as a model for an *in vivo* structure-function analysis. This has allowed a physiological discrimination between the two structural models for how NCS-1 interacts with its target proteins.

## Results

### Role of NCS-1 in temperature-dependent locomotion in *C. elegans*

**Figure 1:**
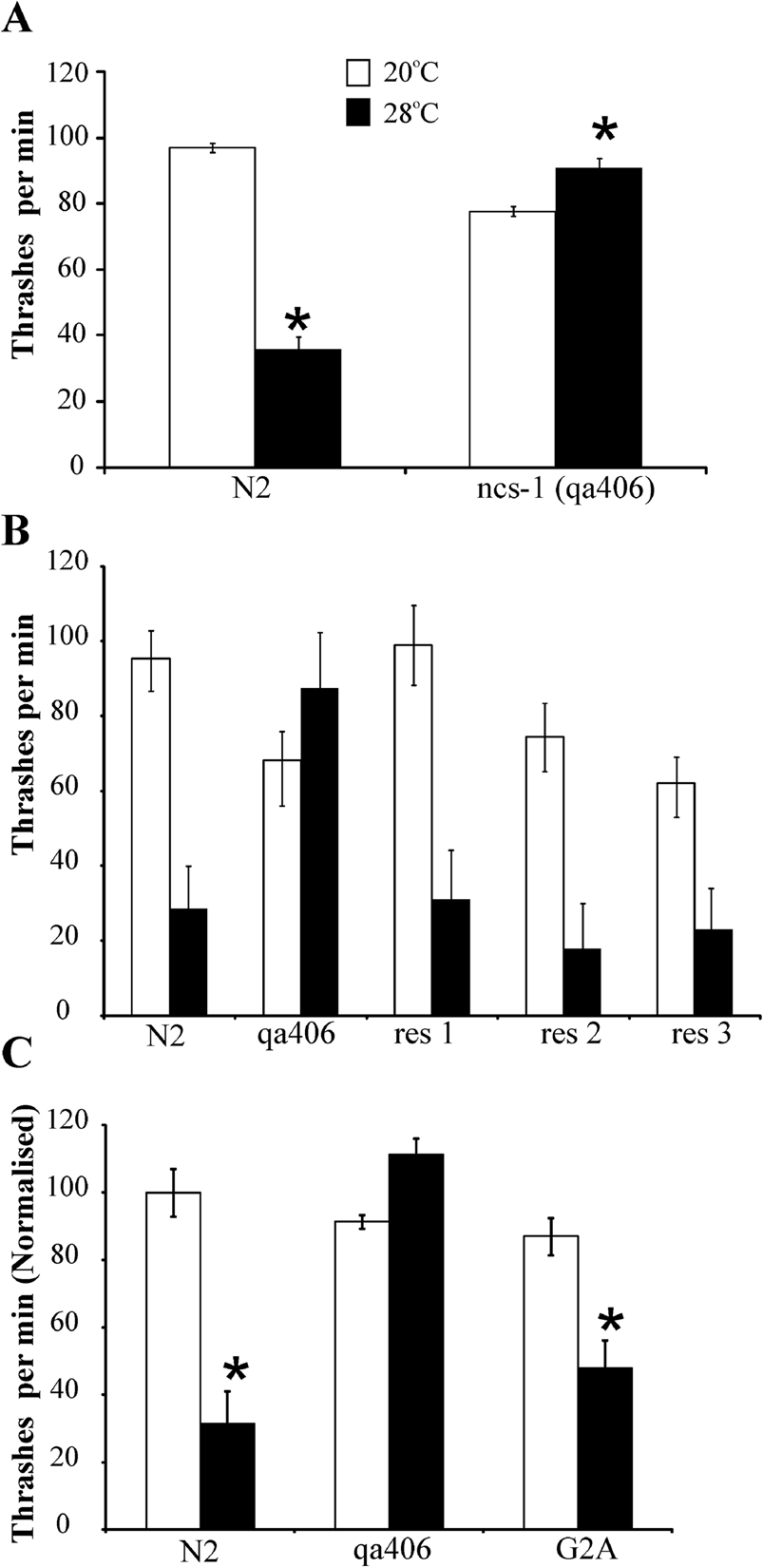
**Loss of function of *ncs-1 *prevents acute inhibition of locomotion following temperature elevation in a temperature-dependent locomotion (TDL) assay. (A)**. Locomotion rates of wild-type Bristol N2 and *ncs-1* null (qa406) worms were quantified at room temperature (20°C; open bars) and then after elevation of temperature to 28°C for 10 min (filled bars). N2 worms showed a substantial reduction in locomotion at elevated temperature (*; P < 0.01) but in contrast the *ncs-1* null worms showed an increase rate of locomotion at 28°C (*; P < 0.01, n = 100 worms for each condition). **(B)** Transgenic expression of NCS-1 rescued the TDL assay phenotype. The locomotion at 20°C and 28°C of three separate lines of transgenic worms expressing NCS-1 under the control of its endogenous promoter (res 1–3) was compared to that of N2 wild-type and *ncs-1* null worms (n = 7-12 for each worm line). **(C)** Non-myristoylatable NCS-1 was able to rescue function in the TDL assay. The locomotion at 20°C and 28°C of three separate lines of transgenic worms expressing NCS-1 (G2A) in the *ncs-1* null background was compared to that of N2 wild-type and *ncs-1* null worms. The data from separate worm lines was pooled and the data normalised to the locomotion rate of N2 worms at 20°C and the rate of movement at 20°C and 28°C compared for each condition (*; P < 0.01, n = 10 for N2 and qa406, n = 30 for G2A). Statistical significance was determined using the Mann–Whitney *U* test with use of the Bonferonni correction for multiple comparisons.

*C. elegans* possesses a single orthologue of human NCS-1. A null *ncs-1* C. elegans mutant (*qa406*) has been shown to be defective in thermotaxis in an assay (isothermal tracking) [47] based on the ability to associate growth temperature with food availability and to move to the preferred temperature within a radial temperature gradient [22]. In contrast, NCS-1 is not required for temperature avoidance behaviour [22,48]. We have recently established a simple assay for temperature-dependent locomotion (TDL) in *C. elegans* as a read out for thermosensory signalling and behaviour based on acute inhibitory effect of elevated temperature on locomotion. Behaviour in the TDL assay and in thermotaxis is effected by distinct genes [49]. The TDL and thermotaxis behaviours are also distinct from temperature avoidance in the worm which involves different genes and neural pathways [50]. The response to temperature in the TDL assay was, however, regulated through the AFD neurons [49] that are within the neural circuit that controls thermotaxis [51]. We determined, therefore, what effect the absence of NCS-1 would have on the response of worms in the TDL assay. At 20°C, wild-type Bristol N2 worms and the *ncs-1* null showed similar coordinated locomotion although the *ncs-1* null moved slightly slower. Acute (10 min) elevation of temperature from 20°C to 28°C resulted in a significant slowing of movement of N2 worms. In contrast, *ncs-1* null worms did not show a depression in locomotion but instead showed a small but significantly faster rate of movement at the elevated temperature (Figure 1A). This is an unusual phenotype that we have not observed in other mutants tested in this assay [49]. To establish that this phenotype was indeed due to the absence of NCS-1 we performed rescue experiments by overexpression of NCS-1 under the control of its endogenous promoter. The data in Figure 1B show the results from a typical single experiment using the TDL assay in which three transgenic lines showed essentially complete rescue of the inhibition of locomotion at 28°C. Since NCS-1 is a myristoylated protein we also examined the effect of transgenic expression of a myristoylation-defective mutant (G2A) [7]. Figure 1C (and subsequent figures) shows data pooled from multiple transgenic lines. In common with functional studies on the yeast orthologues of NCS-1 [11,52], myristoylation did not appear to be essential for functional rescue.

**Figure 2:**
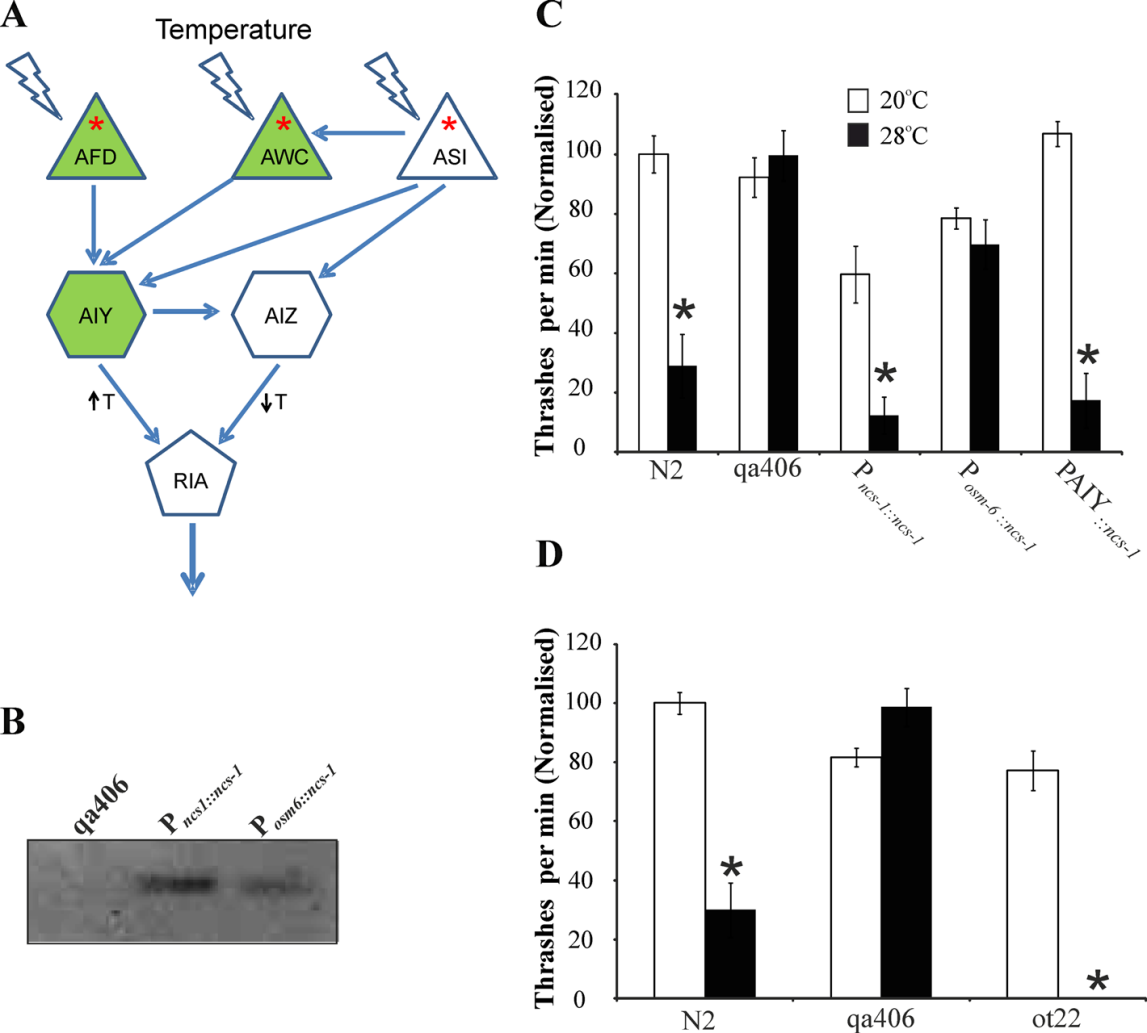
**NCS-1 functions in AIY neurons in the temperature-dependent changes in locomotion. (A)** The circuit that controls thermotaxis in *C. elegans*[55] including information on additional thermosensory neurons [56]. Temperature is sensed by AFD, AWC and ASI neurons, which signal through AIY and AIZ interneurons. Signalling from AIY to RIA results in thermophilic drive whereas signalling from AIZ to RIA results in cryophilic drive. The neurons in green express NCS-1 [22]. Asterisks indicate sensory neurons where expression is driven by the *osm-6* promoter. **(B)** Western blot of *ncs-1* null and transgenic worms with *ncs-1* expression driven by P*ncs-1* or P*osm-6* probed with anti-human-NCS-1. **(C)** Expression of NCS-1 in AIY interneurons rescued the TDL assay phenotype in the *ncs-1* null (qa406). Transgenic strains using different promoters to drive *ncs-1* expression were generated by injection of expression plasmids derived from genomic *ncs-1*. Locomotion rates of wild-type N2 and *ncs-1* null worms were quantified at room temperature (20°C; open bars) and after elevation of temperature to 28°C for 10 min (filled bars). N2 worms showed a reduction in locomotion at elevated temperature (*; P<0.01) not seen with the *ncs-1* null worms. The locomotion at each temperature of three lines of transgenic worms for each condition expressing NCS-1 under the control of indicated promoter was assayed. The data from worm lines were pooled and normalised to the locomotion rate of N2 worms at 20°C and the rate of movement at 20°C and 28°C compared (*; P<0.01, n=15 except for PAIY_::*ncs-1*_ where n=45 worms). **(D)** The *ttx-3* mutant worms show increased inhibition of locomotion at 28°C. Locomotion rates of wild-type Bristol N2 and *ncs-1* null and *ttx-3* mutant worms were quantified at room temperature (20°C; open bars) and then after elevation of temperature to 28°C for 10 min (filled bars) (*; P<0.01, N=20 worms).

The neural circuit that control thermotaxis in *C. elegans* is shown in Figure 2A. As indicated, NCS-1 is known to be expressed in three of these neurons, the AFD, AWC and AIY neurons [22]. The thermotaxis defect in the *ncs-1* null mutant can be rescued by expression in only the AIY neurons. To test if this would also be the case for the TDL assay, transgenic worms were generated with NCS-1 expressed under the control of the *osm-6* promoter that drives expression in multiple sensory neurons including the AFD and AWC neurons but not in the AIY interneurons. Transgenic worms were also generated with NCS-1 expressed using the specific PAIY promoter [53,54]. These were compared to worms expressing NCS-1 under its endogenous promoter in the TDL assay. As shown in Figure 2C, P_*osm-6::ncs-1*_ worms did not show any rescue. This was not a consequence of a general lack of expression of the protein in these worms as this could detected by Western blotting although it was apparently lower than with the ncs-1 promoter (Figure 2B). We have determined that this promoter drives expression of a reporter construct in the appropriate neurons. We cannot formally rule out, however, that expression from this promoter was not at a level sufficient for rescue in certain key neurons but this is unlikely as this represented a significant overexpression of the protein in these worms. In contrast, PAIY*:ncs-1* worms expected to express NCS-1 specifically in the AIY neurons showed full rescue of the phenotype and were indistinguishable from N2 worms in the TDL assay (Figure 2C).

These results suggest that NCS-1 expression in AIY neurons is required and is sufficient for the acute inhibition of locomotion at elevated temperature. To test this further the requirement for functional AIY neurons in the TDL assay was assessed. The transcription factor *ttx-3* is required for the late differentiation of these neurons and this has allowed the role of AIY neurons in chemotaxis to been demonstrated in *ttx-3* mutant worms [53,57,58]. If NCS-1 had a positive effect on signalling in AIY neurons then the *ttx-3* mutant should show a similar phenotype to the *ncs-1* null in the TDL assay. In fact, the opposite was the case with the *ttx-3* mutant (ot22) showing an enhanced sensitivity to elevated temperature (Figure 2D) with essentially complete inhibition of locomotion at 28°C (significantly different from N2 worms at 28°C; p < 0.01). This would be consistent with the idea that NCS-1 acts in AIY neurons and that the role of NCS-1 is to inhibit neuronal activity within the AIY neurons to dampen the pathway that would normally drive movement at elevated temperature.

### Effect of specific mutations within the hydrophobic groove on NCS-1 function

**Figure 3:**
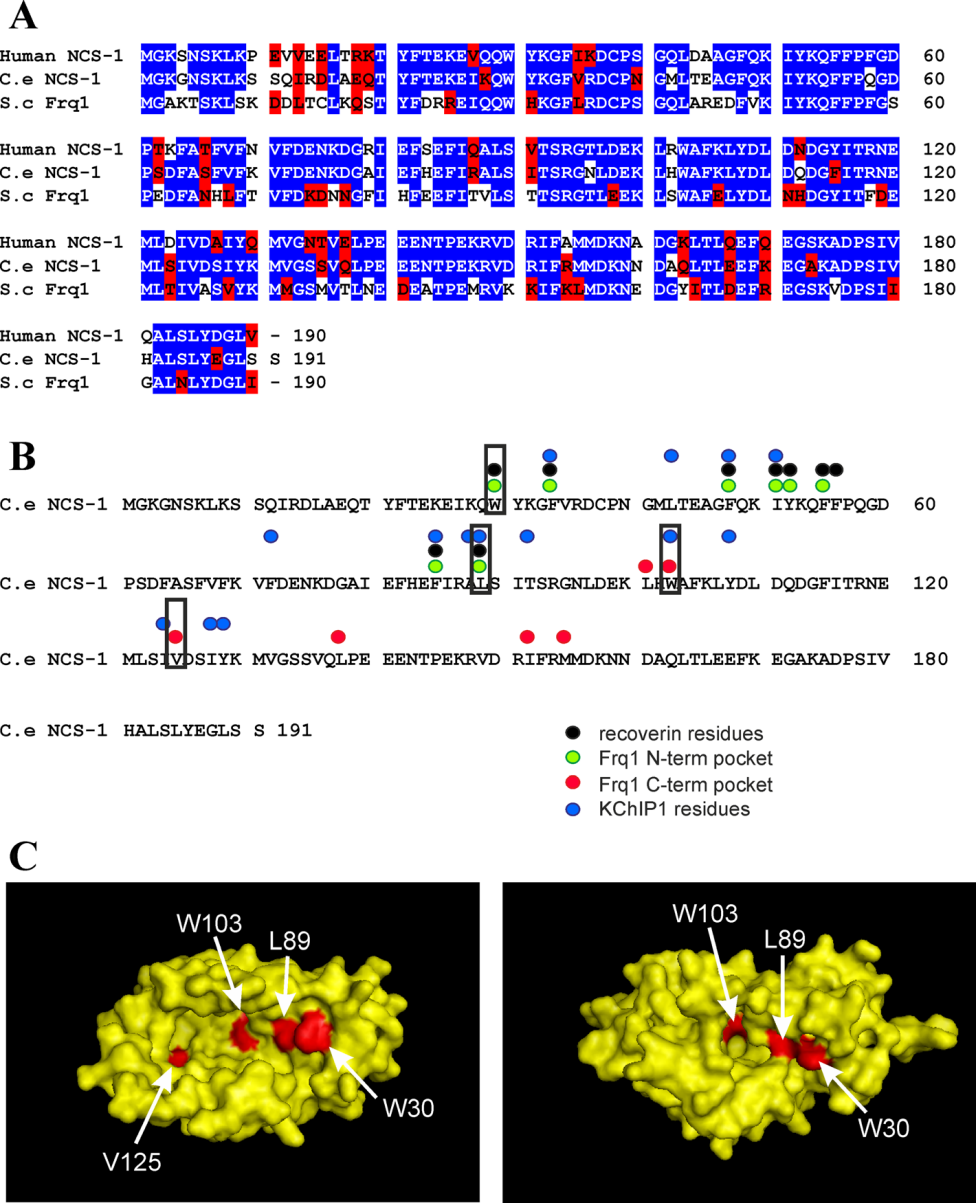
**Comparison of NCS-1 orthologues and identification of residues for targeted mutation. (A)** There is extensive homology between human, *C. elegans* and *S. cerevisiae* NCS-1 orthologues. Identical residues in all three species are shaded in blue and those with similarity across species are shaded in red. **(B)** Hydrophobic residues implicated in target protein interactions are conserved in *C. elegans* NCS-1. Then residues that directly make contact with target proteins in structurally characterised complexes are indicated above the sequence of *C. elegans* NCS-1. Residues selected for mutagenesis are boxed. **(C)** Position of residues selected for mutagenesis in the predicted NCS-1 structure. The selected amino acids are within the hydrophobic groove are shown in red in a surface representation of model structures for *C. elegans* NCS-1 based on the crystal structure of human NCS-1 (PDB1G8I, left) or the solution NMR structure (PDB 2LCP, right). In the structure predicted from the NMR structure V125 is not surface exposed but buried beneath the C-terminal tail and so not visible in the figure.

These results indicated a functional role for NCS-1 in thermosensory behaviour that can be readily assayed based on sensitivity to an acute temperature elevation in the TDL assay and that overlapped with a characterised neural circuit for thermosensation. This provided the basis for subsequent use of the assay for testing key aspects of predictions from structural analyses and examining the physiological relevance of modes of target interaction. *C. elegans* has seven genes encoding NCS proteins (NCS-1-7) with the worm NCS-1 being the orthologue of the human (74.8% sequence identity) and yeast (58.1% identity) proteins (Figure 3A). The hydrophobic residues implicated in direct interaction with target peptides from structural studies are fully conserved across these three species and include residues implicated in target binding in Frq1, recoverin and KChIP1 (Figure 3B). We selected for mutation four residues within the hydrophobic groove (Figure 3B indicated with black boxes). Two (W30 and L89) within the N-terminal pocket of the hydrophobic groove were structurally identified as being directly involved in target binding in all solved complex structures. Two (W103 and V125) were selected from the C-terminal pocket of the hydrophobic groove. V125 has been implicated in direct interaction with Pik1 in the complex with Frq1 [42,43] but in the target peptide complexes made by recoverin and KChIP1 it is not available for target binding due to the position of the C-terminal tail of each NCS protein which makes V125 inaccessible [38,39,41]. These residues are shown on predicted structures of *C. elegans* NCS-1 (Figure 3C). One is based on the crystal structure of human NCS-1 in which the hydrophobic groove is opened out due to bound polyethylene glycols within the crystals [40]. The other on the solution NMR structure in which the C-terminal tail occludes the C-terminal pocket of the hydrophobic groove [46] showing the lack of accessibility of V125 in the NMR structure.

**Figure 4:**
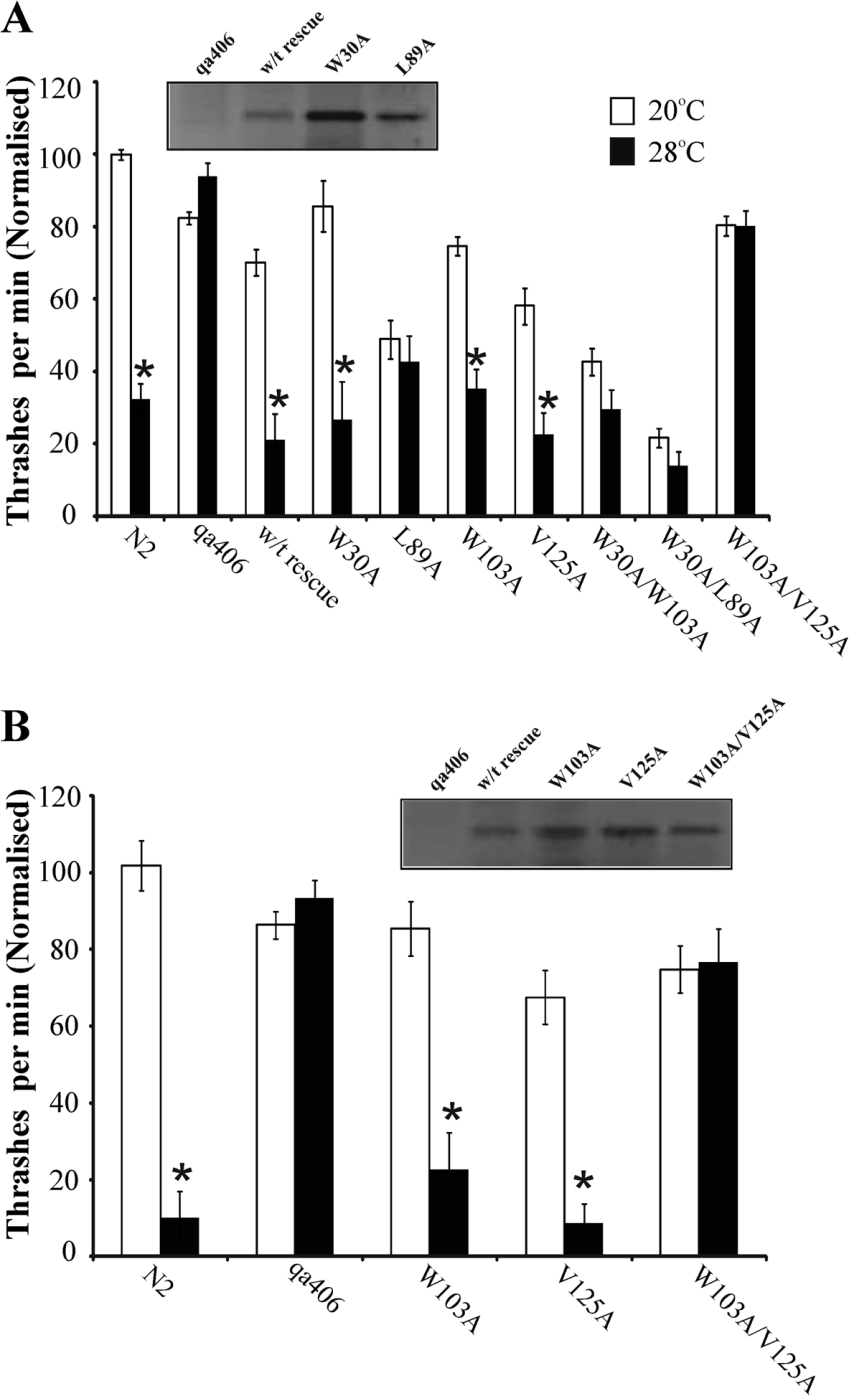
**Effect of single and double mutations on the ability of expressed NCS-1 to rescue the temperature-dependent changes in locomotion. (A)** In each assay locomotion rates of wild-type Bristol N2 and *ncs-1* null (qa406) worms were determined alongside wild-type or mutant expressing transgenic worms at room temperature (20°C; open bars) and then after elevation of temperature to 28°C for 10 min (filled bars). For each condition locomotion at 20°C and 28°C of three separate lines of transgenic worms expressing NCS-1 or its mutants on the *ncs-1* null background was assayed, the data from separate worm lines were pooled and the data from experiments on different days normalised to the locomotion rate of N2 worms at 20°C in that particular assay. The rate of movement at 20°C and 28°C was compared for each condition (*; P < 0.01, n = 28-84 worms). The inset shows a Western blot probed with anti-human-NCS-1of *ncs-1* null worms and transgenic worms with *ncs-1* bearing the indicated mutations. **(B)** Within the same assay the locomotion of N2, *ncs-1* null and transgenic worms expressing W103A, V125A or W103A.V125A was directly compared at room temperature (20°C; open bars) and then after elevation of temperature to 28°C for 10 min (filled bars). The rate of movement at 20°C and 28°C was compared for each condition (*; P < 0.01, n = 15 worms). Statistical significance was determined using the Mann–Whitney *U* test with use of the Bonferonni correction for multiple comparisons**.** The inset shows a Western blot probed with anti-human-NCS-1of *ncs-1* null worms and transgenic worms with *ncs-1* bearing the indicated mutations.

Three transgenic strains expressing each *ncs-1* mutation were tested in the TDL assay in direct comparison with N2 and *ncs-1* null worms. The data from the multiple lines and assays were normalised to the locomotion mean for N2 worms at 20°C and combined for comparison of all mutants (Figure 4A). Locomotion at 28°C was statistically compared to that at 20°C for each mutation. In comparison with rescue of null worms with wild-type NCS-1 it was clear that NCS-1bearing single mutations W30A or W103A showed rescue of the TDL phenotype. In contrast, the L89A mutation in the N-terminal pocket of the hydrophobic groove prevented rescue even though protein expression could be shown by Western blotting (Figure 4A) indicating the potential importance of this part of the groove for function. Worms with the double mutation W30A/W103A also did not show apparent rescue. With both L89A and W30A/W103A expressing worms, locomotion at 20°C was already reduced to 40% of that of N2 worms. This would not be likely to have hidden an effect of elevated temperature in reducing locomotion as such as a temperature-dependent reduction in locomotion was still observed in slow moving lines in a previous study [49] and was also observed in this study in worms with a similarly recorded locomotion at 20°C (not shown). In contrast, in the case of the double mutant W30A/L89A the much lower rate of locomotion of these worms (20% of the rate of N2 worms at 20°C) could have masked any potential rescue.

The results from the mutations in the C-terminal pocket were of particular interest as single mutations (V125A and W103A) did not impair rescue whereas the double mutation (W103A/V125A) abolished the rescue. To verify the difference between single and the double mutations worms expressing these were assayed together at the same time to allow direct comparison and similar results were obtained (Figure 4B). The lack of rescue by NCS-1 bearing the W103A/V125A mutations was not due to absence of expression of the protein (Figure 4B). These results suggest that individual mutations in the C-terminal pocket were not sufficient to reduce target binding on their own but could so when introduced together and would be consistent with the whole of the C-terminal pocket of the hydrophobic groove being required for functional rescue in the TDL assay.

### Effect of C-terminal deletions on NCS-1 function

One interpretation of the results from the single and double mutations is that, in addition to the N-terminal pocket of the hydrophobic groove, exposed hydrophobic residues in the C-terminal pocket of the hydrophobic groove of NCS-1 are functionally required for target interaction. Results from the single and double mutations indicate that V125 acts in concert with W103. In the binding of Pik1 to Frq1 within a complex a conformational change occurs to move the C-terminal tail and to fully expose the hydrophobic groove [42]. In the solution NMR structure for human NCS-1 the C-terminal pocket of the hydrophobic groove is occupied by the C-terminal tail in the absence of ligand [46]. Moreover, characterisation of a point mutant in human NCS-1 (R102Q) identified in a case of autism spectrum disorder [59] revealed that the mutant had structural changes indicating increased dynamics of residues 169–190 [24] and bound target peptide with higher affinity [44] consistent with the C-terminal pocket no longer being occluded by the C-terminal tail in this mutant. This along with the data above from specific hydrophobic residues suggests a mode of interaction of NCS-1 with targets involving the full hydrophobic groove in which the C-terminal tail would not be required for direct interaction with the target and could be functionally dispensable for activation of NCS-1 signalling. To test this possibility, transgenic worms were generated expressing NCS-1 with varying sizes of C-terminal deletion.

**Figure 5:**
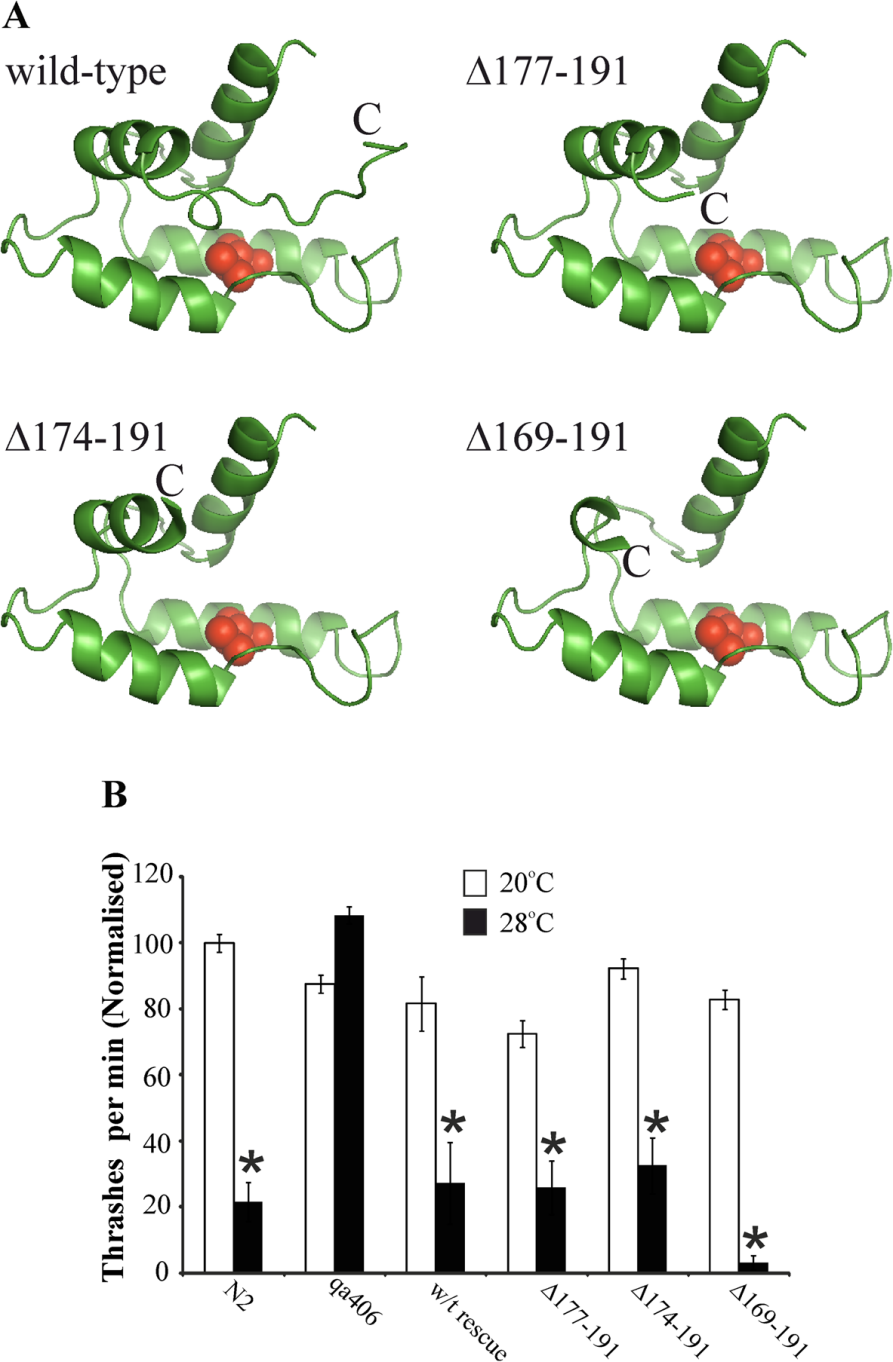
**NCS-1 with deletion of its C-terminal tail is still able to rescue the temperature-dependent changes in locomotion. (A)** Model structure of *C. elegans* NCS-1 derived from the solution NMR structure of human NCS-1(2LCP) indicating the C-terminal deletions tested in this study. The figure shows the C-terminal part of NCS-1 (from residue 95) with the C-terminal pocket of its hydrophobic groove occupied by its C-terminal tail and with the C-terminus indicated by C. The tail and increasing amounts of the preceding helix are deleted in the mutants ∆177-191, ∆174-191 and ∆169-191. V125 is shown in red. **(B)** Locomotion rates of wild-type Bristol N2, *ncs-1* null (qa406), wild-type rescue or C-terminal deletion expressing transgenic worms were assayed at room temperature (20°C; open bars) and then after elevation of temperature to 28°C for 10 min (filled bars). For each deletion mutant locomotion of three separate lines of transgenic worms was assayed, the data from separate worm lines pooled and the data from experiments on different days normalised to the locomotion rate of N2 worms at 20°C in that particular assay. The rate of movement at 20°C and 28°C was compared for each condition (*; P < 0.01, n = 15-30 worms). Statistical significance was determined using the Mann–Whitney *U* test with use of the Bonferonni correction for multiple comparisons.

Rather than simply test a single mutant with a large deletion (∆169-191) reflecting the R102Q mutant we also tested smaller deletions in case the large deletion had deleterious effects on the protein. The mutant with the smallest deletion (∆177-191) was based on minimal removal of those residues within the C-terminal tail that interact within the C-terminal pocket of the hydrophobic groove. NCS-1 ∆177-191 would have lost the C-terminal loop up to the preceding α-helix so that the C-terminal hydrophobic pocket (including residue V125) would be largely exposed (Figure 5A). The two larger deletions that were also tested would not only remove this C-terminal tail but also residues within the preceding helix. As shown in Figure 5B, expression of either ∆177-191or ∆174-191 was able to rescue fully the TDL phenotype and the worms were indistinguishable from the wild-type rescue in the assay. Interestingly, worms expressing the larger deletion (∆169-191) were not only rescued but showed greater inhibition of locomotion at 28°C than N2 worms consistent with a gain of function. These results show that the extreme C-terminal tail of NCS-1 is not required for function and is presumably therefore not crucial, in a physiological context, for target binding.

## Discussion

We have established an assay using the model organism, C. elegans to allow the study of structure-function aspects of NCS-1 signalling in an *in vivo* situation. Using the TDL assay we have shown an essential role for NCS-1 in regulation of temperature-dependent locomotion in *C. elegans* that requires expression of NCS-1 only in the AIY neurons. These neurons have previously been implicated in thermotaxis [60] although this behaviour differs from the TDL assay we have used. Nevertheless, NCS-1 in AIY neurons is required for both thermotaxis and TDL behaviour but is not involved in the temperature-avoidance response behaviour [22,48]. Using the TDL assay, we have carried out a structure-function analysis to allow a physiological test of the structural basis for NCS-1 interaction with its target proteins. We have used the worm model to assess the functional relevance within a behavioural assay of key identified residues within the N- and C-terminal pockets of the hydrophobic groove of NCS-1 and the relevance of its extreme C-terminal tail. The results are consistent with the notion that the C-terminal tail of NCS-1 is not essential whereas the full length of the hydrophobic groove is likely to be involved in regulatory interactions required for function.

Previous work demonstrated NCS-1 expression in 10 pairs of sensory neurons including the AIY neurons, two pairs of interneurons, one motor neuron and one muscle cell type based on use of a GFP reporter construct [22]. This pattern of expression was confirmed using immunohistochemistry that showed overlap of GFP and antibody staining [61]. Notably functional rescue was achieved in the previous [22] and the current study following expression in only the AIY neurons and so the functional role of NCS-1 in other cell types remains to be established. It was previously believed that C. elegans harboured three NCS-1 family members [3] but a recent reanalysis of neuronal genes has identified a further four [62]. It is not known where NCS-1 and NCS-2 are expressed. Deletion of *ncs-2* is lethal whereas the *ncs-3* null has no detectable phenotype [63]. The sites of expression and functions of the other NCS proteins is unknown. The seven worm NCS proteins are more divergent as a family than the mammalian NCS proteins. The sequence identity between the worm NCS-1 and the other worm NCS proteins ranges from as low as 9.7 to 59.9% compared to the human proteins that are 24.6-58.0% identical to human NCS-1. The worm NCS proteins also have distinct C-terminal sequences that will likely affect target interactions. It is likely, therefore, that the worm NCS proteins will have divergent functions from that characterised for NCS-1. Considerable further work will be required for an understanding of the functions of the other NCS proteins.

Mammalian NCS-1 has non-redundant functions not compensated for by other family members [3,10]. NCS-1 is known to interact with a wide range of mammalian target proteins [25,26] including PI4KIIIβ [11,14,27], ARF1 [14,28], IL1RAPL1 [24,29], TRPC5 channels [18], InsP(3) receptors [30] and dopamine D2 and D3 receptors [31,44]. Its direct targets in *C. elegans* have not been determined although orthologues of many of the above proteins are present in this organism. The specific and non-redundant functions of NCS-1 and other NCS proteins may reside in part in their distinct subcellular localisation and membrane associations and on neuron-specific patterns of expression and will be determined by their interactions with specific target proteins [3,10]. Many such targets have been identified which can be highly specific for one or other NCS protein. NCS proteins have very similar overall topology [33] and they all have an exposed hydrophobic groove in the Ca^2+^ bound form [6,34-40]. Specificity of target interaction has been suggested to be due to the varying size and shape of the hydrophobic groove, differences in surrounding charged residues and interactions of the extreme C-terminus of the proteins [10,33]. Structural data is available for complexes of recoverin with rhodopsin kinase [41], KChIP1 with the Kv4.3 potassium channel [38,39] and orthologues of NCS-1 (Frq1) in yeasts with Pik1 [42,43]. In addition, the interaction of NCS-1 with a peptide derived from the C-terminus of the dopamine D2 receptor has been characterised [44]. In each case the target peptide(s) was bound within a solvent-exposed hydrophobic groove in the Ca^2+^-bound form of the NCS protein. In the case of the KChIP1/Kv4.2 interaction there was also an additional interaction site on the outside of the second N-terminal helix of KChIP1 [38,39]. The hydrophobic groove is formed from hydrophobic residues conserved in all of the NCS family members suggesting that a similar mode of target binding could be used by all of the NCS proteins. However, the exact topological relationship of the target helix with the NCS protein and the size of the exposed part of the hydrophobic groove varied between complexes [33] with this being predominately based in an N-terminal pocket or both N- and C-terminal pockets within the groove. An important feature contributing to specificity of target interaction is the variable C-terminal sequence of the NCS proteins. In the Frq1/Pik1 complexes [42,43] and in the KChIP1/KV4.3 complex [38,39] the C-terminal helix appeared to be rotated out in the Ca^2+^ bound form to expose aspects of the hydrophobic groove whereas in the case of recoverin the C-terminal helix occupied the C-terminal pocket of the groove in the complex [41]. In addition, the C-terminal tail of recoverin was required due to its direct interactions with the target peptide and was necessary for high affinity interaction with rhodopsin kinase [45]. In the NMR structure of NCS-1 the C-terminal tail was seen to occupy the C-terminal part of the hydrophobic groove (Figure 3C) in the absence of ligand [46] potentially preventing ligand interaction with the C-terminal pocket of the groove. A mutation that was discovered in a case of autistic spectrum disorder [59] affected the C-terminal domain and the potential exposure of the C-terminal pocket of hydrophobic groove [24,46]. Whether or not the C-terminal tail of NCS-1 is directly involved in target binding or instead needs to undergo a conformational change to expose the full length of the hydrophobic groove has remained to be resolved but we have now addressed this question in an *in vivo* context.

The structural studies outlined above have provided clues to the factors that determine the specificity of target binding that could in turn underlie the different physiological function of the NCS proteins. Nevertheless, a major issue is that all of the studies were based on the analysis of the interactions with only short peptides derived from the target proteins. It remains possible, therefore, that within the constraints of a full-length protein the domains may interact with NCS proteins by alternative mechanisms. In addition, the two published structures for human NCS-1 differ to the extent that that are each most consistent with one or other of the proposed models for target interaction [40,44,46]. It is important, therefore, that the key aspects identified in structural studies are tested in functional assays within intact systems and *C. elegans* has provided a model system to assess structure-function aspects of NCS-1 signalling.

Previous studies in yeast have shown that myristoylation of NCS-1 is not essential but reduces the efficiency of rescue [11,52]. The G2A mutation prevents myristoylation [7,64] but NCS-1 bearing this mutation was able to rescue function in the *ncs-1* null mutant. This indicates that membrane targeting through the myristoyl group is not essential for NCS-1 function. This may be a consequence of levels of overexpression of the NCS-1 in the transgenic worms with membrane targeting only being important for efficient function of NCS-1 at endogenous expression levels. One important consequence of this result, however, is that it indicates that NCS-1 must function by regulation of a cytosolic or already membrane bound target and its role cannot primarily be the recruitment of bound proteins to membranes.

The lack of ability to rescue in the *ncs-1* null mutant by other specific NCS-1 mutations compared to G2A suggests that these other residues must be particularly crucial for NCS-1 function. Mutation of L89 and the double mutation W103A/V125A failed to rescue in the TDL assay suggesting that interactions across both N- and C-terminal pockets of the hydrophobic groove are functionally important for NCS-1 in the worm. The lack of effect on function of NCS-1 of the single mutations W103A and V125A in contrast to the lack of rescue by the double mutant suggests that both of these residues contribute to the affinity of target protein interaction. Fully consistent with results from these specific mutations was the finding that truncation of the C-terminus of NCS-1 to remove between 14 and 22 C-terminal residues did not impair its ability to rescue. This is in marked contrast with analysis of recoverin where C-terminal truncation of as few as 7 residues had a marked inhibitory effect on its affinity for rhodopsin kinase [45]. We cannot rule out from these studies that the C-terminal tail of NCS-1 may play a regulatory auto-inhibitory role by switching off NCS-1 function or in preventing inappropriate binding interactions due to its ability to bind within the C-terminal pocket [46], but is clearly not required for NCS-1 to be effective in the TDL assay. The finding that the full length of the hydrophobic groove is likely to be involved in functionally relevant target interactions is consistent with published structures of the yeast orthologues of NCS-1 with bound target peptides [42,43].

## Conclusions

The current work has allowed physiological assessment of suggestions from structural studies on the key structural features that underlie the interaction of NCS-1 with its target proteins in an *in vivo* context. We have established a key physiological role for hydrophobic residues within the Ca^2+^-exposed hydrophobic groove of NCS-1 that mediate structural interactions with target peptides. Importantly, the results have shown that the C-terminal tail of NCS-1 is not functionally required allowing discrimination between the two potential modes of interaction of NCS-1 with its target proteins.

## Materials and methods

### Plasmids

The marker plasmid pRAB100 [P_*rab-3*_::GFP] was a gift from the Nonet Lab (Washington University of St Louis, USA) and contains the EGFP reporter protein gene driven by the *C. elegans rab-3* pan neuronal promoter. The PAIY::MCS plasmid [53] was a gift from the Hobert Lab (Columbia University Medical Center, N.Y.) and contains a promoter from within the introns and exons of the *ttx-3* gene on chromosome X to drive the specific expression of transgenic proteins in the AIY neurons.

### Nematode culture and strains

*C. elegans* were grown and maintained on standard nematode growth medium (NGM) (50 mM NaCl, 1 mM CaCl_2_, 1 mM MgSO_4_, 25 mM KH_2_PO_4_, 5 μg/ml cholesterol, 0.25% w/v peptone, 2% w/v agar) on 60 mm petri plates *E. coli* OP50 strain as the single food source as described previously [65] and kept at 20°C. To age synchronise *C. elegans* strains, 5–10 young healthy hermaphrodite adult worms were transferred, as above, to seeded plates, left for ~ 16 hr to lay eggs, then removed and the eggs and larva were incubated for ~36 hrs, ~ 60 hrs or ~84 hrs for progeny to hatch and mature to approximately stage 4 larval/Day 0, Day 1 or Day 2 adults respectively. The wild-type reference strain was Bristol N2 and mutant alleles used in this study were *ncs-1* (qa406) and *ttx-3* (ot22) obtained from the *Caenorhabditis* Genetics Centre (CGC) (University of Minnesota, USA).

### Expression constructs and generation of transgenic animals

Transgenic strains were generated by germline injection. Injection plasmids were constructed using standard molecular biological techniques as performed previously [66,67]. Transgenic rescues using *ncs-1* were performed either using the genomic *ncs-1* gene sequence (Wormbase gene sequence C44C1.3, accession number CCD63971) or synthetically generated *ncs-1* coding sequence in each case driven by the endogenous *ncs-1* promoter. The *ncs-1* gene promoter sequence was defined as stretching 3.5 kbp from before the start sequence of the *ncs-1* gene as previously reported [22]. Plasmids containing synthetic spliced (without any introns) wild type and mutated *ncs-1* genes were purchased from Geneart (Life technologies). Transgenic strains were also generated by injection of plasmids incorporating genomic ncs-1 under the control of *osm-6* and AIY promoters.

For injection, larval stage 4 to Day 1 adult N2 or *ncs-1* null worms were injected with expression constructs into the germ line cells of the dorsal gonad. The injection mixtures contained DNA concentrations of 100 ng/μl DNA consisting of 10 ng/μl *ncs-1* expressing plasmid, 40 ng/μl of GFP marker plasmid ([P_*rab-3*_::GFP] or [P_osm-6_::GFP]) and 50 ng/μl of empty plasmid in injection buffer (20 mM KPO_4_, 3 mM Citrate, 2% (w/v) PEG 6000). Worms were immobilised on a 2% (w/v) agarose pad on a glass coverslip and coated with halocarbon oil. The worms were visualised for injection using Nikon Eclipse Ti-S inverted microscope at 40x and an Eppendorf micromanipulator was used to position the needle. After injection, the worms were rehydrated with M9 buffer and moved to a seeded NGM plate. Injected plates were observed for GFP expression in progeny. F1 generation worms were isolated on to individual plates. Expression of GFP in the F2 generation confirmed incorporation of the extra chromosomal plasmids in the worm. Three separate lines for each expression construct were generated and found to display the same phenotypes.

### Temperature dependent locomotion assay

The temperature dependency of locomotion was assessed by measuring the thrashing rate of the worms at 20°C and 28°C for wild type and mutant strains. A Petri dish containing 200 μl of Dent’s solution (140 mM NaCl, 6 mM KCl, 1 mM CaCl_2_, 1 mM MgCl_2_, 5 mM HEPES, pH 7.4 with bovine serum albumin at 0.1 mg/ml) was placed on the centre of a Peltier effect thermoelectric plate. The temperature of the Dent’s solution was monitored by a thermocouple and the temperature was recorded in real time. A day 1 or day 2 adult worm was removed from an NGM plate and immersed in the Dent’s solution at 20 ± 0.3°C. The worm was left to acclimatise for 10 minutes then the thrashes per minute were counted. A single thrash was defined as a complete change direction of bending of the mid body then back again to the original position. The droplet of Dent’s solution was then heated up to 28 ± 0.3°C, the worm was left for a further 10 minutes to acclimatise and the thrashing rate was recorded at the higher temperature. At least 15 worms were assayed for each of the strains. All data were expressed as means ± S.E.M. Where indicated, the data were pooled and normalised to the thrashes per minute for the N2 wild type strain at 20°C. The significance of the effect of mutations on locomotion rates at 28°C compared to 20°C was assessed using the Mann–Whitney *U* test and the error rate for multiple comparisons was controlled using the Bonferroni correction.

### C. elegans protein extraction and Western blotting

To extract proteins from the worms, 35 whole animals were placed in 25 μl 4% SDS loading buffer for each strain and frozen at −80°C overnight. The sample was then heated to 100°C for 20 minutes and the proteins separated by SDS-PAGE electrophoresis on a 4-12% Bis-Tris polyacrylamide gel. The proteins were transferred to a nitrocellulose membrane and probed using polyclonal rabbit anti-human-NCS-1 antibody [13] at a dilution of 1:1000.

### Protein structure prediction and rendering

Predicted models of *C. elegans* NCS-1 structure were generated from its amino acid sequence using the SWISS-MODEL server [68] based on the published crystal (PDB entry 1B8I) [40] or solution NMR structures of human NCS-1 (PDB entry 2LCP) [46].The three-dimensional representations of the structures were produced in PyMol (Delano Scientific).

## Abbreviations

GCAPs: Guanylate cyclase activating proteins; IL1RAPL1: Interleukin receptor accessory protein like-1; KChIPs: K^+^ channel interacting proteins; NCS: Neuronal calcium sensor; PI4K: Phosphatidylinositol-4-kinase; TDL: Temperature-dependent locomotion.

## Competing interests

The authors declare that they have no competing interests.

## Authors’ contributions

VVM and JRJ performed the experiments. RDB conceived the experiments. VVM, LPH, JWB and RDB contributed to analysis and interpretation of the data. LPH, JWB and RDB wrote the paper. All authors read and approved the final manuscript.
